# Diamidines *versus* Monoamidines as Anti-*Pneumocystis* Agents: An *in vivo* Study

**DOI:** 10.3390/ph6070837

**Published:** 2013-07-01

**Authors:** Dimitri Stanicki, Muriel Pottier, Nausicaa Gantois, Claire Pinçon, Delphine Forge, Isabelle Mahieu, Sébastien Boutry, Jean Jacques Vanden Eynde, Anna Martinez, Eduardo Dei-Cas, El-Moukhtar Aliouat

**Affiliations:** 1 Laboratory of Organic Chemistry, University of Mons-UMONS, B-7000 Mons, Belgium; E-Mails: dimitri.stanicki@umons.ac.be (D.S.); delphine.forge@umons.ac.be (D.F.); 2 Biology & Diversity of Emerging Eukaryotic Pathogens (BDEEP), Center for Infection and Immunity of Lille (CIIL), INSERM U1019, CNRS UMR 8204, EA-4547, Univ Lille Nord de France, UDSL, Institut Pasteur de Lille, F-59000 Lille, France; E-Mails: muriel.pottier@univ-lille2.fr (M.P.); nausicaa.gantois@pasteur-lille.fr (N.G.); mtz.anna@gmail.com (A.M.); eduardo.deicas@gmail.com (E.D.-C.); elmoukhtar.aliouat-3@univ-lille2.fr (E-.M.A.); 3 EA2694, Department of Biostatistics, Université de Lille Nord de France, F-59000 Lille, France; E-Mail: claire.pincon@univ-lille2.fr; 4 Department of General, Organic, and Biomedical Chemistry, NMR and Molecular Imaging Laboratory, University of Mons-UMONS, B-7000 Mons, Belgium; E-Mails: isabelle.mahieu@umons.ac.be (I.M.); sebastien.boutry@umons.ac.be (S.B.); 5 CHU Lille, Biology & Pathology Center, Parasitology-Mycology, F-59000 Lille, France

**Keywords:** *Pneumocystis*, pentamidine, diamidine

## Abstract

Some compounds articulated around a piperazine or an ethylenediamine linker have been evaluated *in vitro* to determine their activity in the presence of a 3T6 fibroblast cell line and an axenic culture of *Pneumocystis carinii*, respectively. The most efficient antifungal derivatives, namely *N*,*N′*-bis(benzamidine-4-yl)ethane-1,2-diamine (compound **6**, a diamidine) and *N*-(benzamidine-4-yl)-*N′*-phenylethane-1,2-diamine (compound **7**, a monoamidine), exhibited no cytotoxicity and were evaluated *in vivo* in a rat model. Only the diamidine **6** emerged as a promising hit for further studies.

## 1. Introduction

*Pneumocystis* fungus lives in the lungs of mammals without developing any risk for healthy individuals. Anarchic proliferation of *Pneumocystis* however is observed in immunosuppressed populations and can induce a severe interstitial lung disease known as *Pneumocystis* pneumonia (PcP) or pneumocystosis [1,2]. That opportunistic illness is widely recognized as one of the initial signs of a HIV infection, but it also affects transplant recipients and patients with malignancies, as well as those receiving chronic corticosteroid therapy or suffering from protein malnutrition, connective tissue diseases, acute lymphatic leukaemia, *etc*. [3]. *Pneumocystis* could also be responsible for upper respiratory tract problems in very young immunocompetent children [4]. Despite its major impact on public health, no continuous *in vitro* model allows to grow *Pneumocystis* for a long period of time, implying that most researches have to rely on animal models of pneumocystosis.

**Figure 1:**
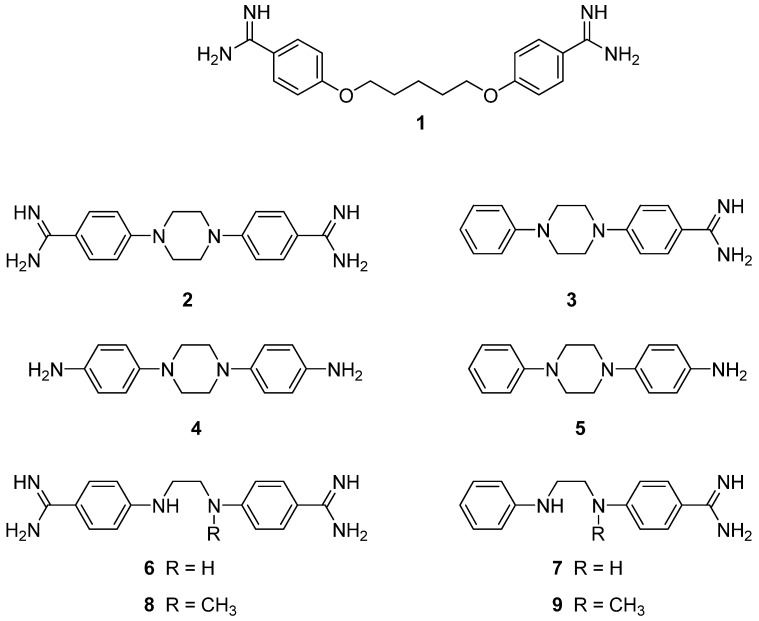
Structure of pentamidine **1** and derivatives **2**–**9** considered in this study.

There are few drugs in clinical use that are effective against *Pneumocystis* [2,5]. The well-known trimethoprim-sulfamethoxazole association constitutes the privileged treatment, but failure occurs in as many as 20 percent of cases. In addition many adverse effects and emergence of sulfa-resistant strains of the fungus have been reported [6,7]. Thus, up to now pentamidine (**1**, Figure 1) has emerged as an alternative to treat moderate to severe *Pneumocystis*-induced pneumonia [2,8]. That molecule exhibits a broad spectrum of antiprotozoal activity: it inhibits the metabolism of *p*-aminobenzoic acid, interferes with anaerobic glycolysis, acts on oxidative phosphorylation, and impairs nucleic acid and protein synthesis [4]. However administration of pentamidine can be followed by numerous side effects [2,9], including immediate reactions (hypoglycemia, nausea, and tachycardia), local reactions (pain, abscess, or necrosis at the sites of injection), and systemic reactions (nephrotoxicity, leucopenia, abnormalities in glucose metabolism).

Pentamidine is a bisbenzamidine in which both benzamidine functions are linked to a highly flexible chain through aryl ether moieties. Many structural modifications have been considered for years in the laboratories of Tidwell and Boykin [10,11,12,13,14] in order to design and synthesize novel diamidines with the hope to identify less toxic and more efficient drug candidates. The topic actually benefits from a renewal of interest as illustrated by a series of recent publications [15,16,17,18]. In previous work [19], we had demonstrated that 4,4′-(1,4-piperazinediyl)bisbenzamidine (**2**, Figure 1) was a promising hit characterized by a marked *in vitro* activity against *P. carinii*. Moreover, we showed [20] that **3** (Figure 1), the monoamidine analogue of **2**, exhibited also an interesting anti-*Pneumocystis* activity, even at submicromolar concentrations. We now extend those studies by evaluating the effect of compounds bearing amino groups (compounds **4**, **5**) instead of amidines and derivatives in which the central rigid piperazine has been replaced by a flexible ethylenediamine (compounds **6**, **7**) or *N*-methyl ethylenediamine (compounds **8**, **9**) linkers.

## 2. Experimental

### 2.1. Chemistry

All compounds were prepared according to previously described procedures [15,19,20]. Their structure was determined on the basis of their IR (Perkin-Elmer FTIR 1760 K) and NMR (Bruker AMX 300 MHz) spectra. Identity and purity of novel compounds were in addition confirmed by HRMS (Waters QToF 2) and elemental analyses (Centre Wallon de Recherches Agronomiques, Libramont-Chevigny, Belgium or Laboratoire de Microanalyse Organique of the Institut des Sciences Appliquées de Rouen, France). Derivatives **2** [19], **3** [20], **4** [21], **6** [22], **8** [22], **10** [23], **11** [24], **12** [25], **13** [22], **14** [22], **15** [22], and **17** [26] have been described in the literature. Compound **16** was commercially available (Aldrich, St. Louis, MO, USA).

*1-(4-Aminophenyl)-4-phenylpiperazine* (**5**). Yield: 57%. White solid. Mp: 146–149 °C. ^1^H-NMR (DMSO-*d_6_*): δ (ppm): 7.23 (2H, t, *J* = 8 Hz); 7.02 (2H, d, *J* = 8 Hz); 6.82 (1H, t, *J* = 8 Hz); 6.75 (2H, d, *J* = 9 Hz); 6.51 (2H, d, *J* = 9 Hz); 4.59 (2H, s, Ar-NH_2_); 3.24 (4H, t, *J* = 5 Hz, CH_2_-N); 3.04 (4H, t, *J* = 5 Hz, CH_2_-N). IR (KBr): ν (cm^−1^): 3359 (N-H), 3220 (N-H), 1600, 1519, 1499. HRMS (ESI-ToF) MH^+^ C_16_H_21_N_4_: exp: *m/z* 254.1651; calc.: *m/z* 254.1657. Elemental analysis C_16_H_19_N_3_: calc.: C 75.85; H 7.56; N 16.59; exp.: C 75.80; H 7.61; N 16.72.

*N-(Benzamidine-4-yl)-N′-phenylethane-1,2-diamine sulfate salt* (**7**). Yield: 28%. White solid. Mp: 191–193 °C. ^1^H-NMR (DMSO-*d_6_*): δ (ppm): 8.92 (2H, s, NH amidine); 8.48 (2H, s, NH amidine); 7.71 (2H, d, *J* = 9 Hz); 7.48 (5H, m); 6.72 (2H, d, *J* = 9 Hz); 3.54 (4H, s, CH_2_-NH-Ar and Ph-NH-CH_2_). IR (KBr): ν (cm^−1^): 3356 (N-H), 3311 (N-H), 1687, 1464, 1215, 853. HRMS (ESI-ToF) MH^+^ C_16_H_18_N_3_: exp: *m/z* 255.1612; calc.: *m/z* 255.1610. Elemental analysis C_15_H_18_N_4_.1.1H_2_SO_4_: calc.: C 49.75; H 5.62; N 15.47; exp.: C 49.49; H 5.70; N 15.49.

*N-(Benzamidine-4-yl)-N-methyl-N′-phenylethane-1,2-diamine hydrochloride salt* (**9**). Yield: 30%. White solid. Mp: 212–215 °C. ^1^H-NMR (DMSO-*d_6_*): δ (ppm): 8.87 (2H, s, NH amidine); 8.72 (2H, s, NH amidine); 7.81 (2H, d, *J* = 9 Hz); 7.07 (2H, t, *J* = 7 Hz); 6.85 (2H, d, *J* = 9 Hz); 6.57 (2H, d, *J* = 7 Hz); 6.50 (1H, t, *J* = 7 Hz); 5.74 (1H, t br., C_6_H_5_-NH); 3.58 (2H, t, *J* = 5 Hz, CH_2_-N(CH_3_)-Ar); 3.21 (2H, t, *J* = 5 Hz, Ph-NH-CH_2_); 3.06 (3H, s, CH_2_-N(CH_3_)-Ar). IR (KBr): ν (cm^−1^): 3289 (N-H), 3123 (N-H), 1661, 1600, 1494, 1390, 1178. HRMS (ESI-ToF) MH^+^ C_16_H_21_N_4_: exp: *m/z* 269.1762; calc.: *m/z* 269.1766. Elemental analysis C_16_H_20_N_4_.HCl: calc.: C 63.05; H 6.94; N 18.38; exp.: C 62.72; H 6.93; N 18.45.

*N-(4-Cyanophenyl)-N′-phenylethane-1,2-diamine* (**18**). Yield: 63%. White solid. Mp: 101–104 °C. ^1^H-NMR (DMSO-*d_6_*): δ (ppm): 7.52 (2H, d, J = 8 Hz); 7.04 (2H, t, J = 7Hz); 6.83 (1H, t br., NH-ArCN); 6.75 (2H, d, *J* = 8 Hz); 6.65 (2H, d, *J* = 7 Hz); 6.52 (1H, t, *J* = 7 Hz); 5.63 (1H, t br., C_6_H_5_-NH); 3.27 (2H, t, *J* = 5 Hz, CH_2_-NH-Ar-CN); 3.20 (2H, t, *J* = 5 Hz, C_6_H_5_-NH-CH_2_). IR (KBr): ν (cm^−1^): 3419 (N-H), 3362 (N-H), 2210 (C≡N), 1603, 1528, 1172. HRMS (ESI-ToF): MH^+^ C_15_H_16_N_3_; exp: *m/z* 238.1342; calc.: *m/z* 238.1344. Elemental analysis C_15_H_15_N_3_: calc.: C 75.92; H 6.37; N 17.71; exp.: C 75.61; H 6.17; N 17.69.

*N-(4-Cyanophenyl)-N-methyl-N′-phenylethane-1,2-diamine* (**19**). Yield: 30%. White solid. Mp: 92–94 °C. ^1^H-NMR (DMSO-*d_6_*): δ (ppm): 7.53 (2H, d, *J* = 8 Hz); 7.12 (2H, t, *J* = 7 Hz); 6.85 (2H, d, *J* = 8 Hz); 6.57 (2H, d, *J* = 7 Hz); 6.49 (1H, t, *J* = 7 Hz); 5.61 (1H, br. t, C_6_H_5_-NH); 3.58 (2H, t, *J* = 5 Hz, CH_2_-N(CH_3_)-Ar-CN); 3.24 (2H, t, *J* = 5 Hz, C_6_H_5_-NH-CH_2_); 3.01 (3H, s, CH_2_- N(CH_3_)-Ar-CN). IR (KBr): ν (cm^−1^): 3394 (N-H), 2210 (C≡N), 1609, 1530, 1516, 1385, 807. HRMS (ESI-ToF): MH^+^ C_16_H_18_N_3_: exp: *m/z* 252.1498; calc.: *m/z* 252.1501. Elemental analysis C_16_H_17_N_3_: calc.: C 76.46; H 6.82; N 16.72; exp.: C 76.42; H 6.86; N 16.68.

### 2.2. Determination of the *in Vitro* Cytotoxicity

*In vitro* cytotoxicity assays with established cell lines are useful tools for the general screening of chemicals in toxicological studies [27]. Thus the cytotoxic effect of the compounds has been evaluated by quantification of cell viability of an adherent 3T6 fibroblast cell line (*In vitro* toxicology assay kit, MTT based, Sigma Aldrich, Bornem, Belgium). The procedure, according to the manufacturer recommendations, was based on the colorimetric determination of the succinate dehydrogenase activity by visualization (540 nm) of the conversion of a tetrazolium salt into formazan [28]. With a microplate reader (Thermo Labsystems Multiskan Ascent 354, Waltham, MA, USA), the optical density of each well was measured at 540 nm against the background absorbance whose reference filter was set at 690 nm. Blank controls (culture medium), free-drug controls, and solvent (DMSO) controls were included in each assay. All cytotoxicity assays were set up in triplicate. The % inhibition of cell proliferation (% IC) was calculated for each compound using the formula: % IC = 100 − [corrected mean OD sample X 100/corrected mean OD solvent controls] where corrected mean OD = mean OD − mean OD of blank controls. The IC_50_ parameters were obtained by plotting % inhibition values against the logarithm of concentration for each compound.

### 2.3. Source of P. Carinii

Athymic *Pneumocystis*-free Lou nu/nu rats (Institut Pasteur de Lille, Lille, France) were used as source of *P. carinii* organisms for all experiments [29]. Dexamethasone (Merck Sharp & Dohme Chibret, France) was administered to ten-week-old female or male nude rats for two weeks in the drinking water (1 mg/L). Then rats were inoculated with 10^7^ of cryopreserved parasites using a non-surgical endotracheal method [30,31,32,33]. Dexamethasone treatment was maintained until the end of the experiment. Six to eight weeks post-inoculation (p.i.) rats were highly infected without secondary fungal or bacterial infection. Animals were housed in HEPA-filtered air isolators (Flufrance, Val de Reuil, France) and were fed with sterile irradiated food (Scientific Animal Food & Engineering [SAFE], Augy, France) and sterile water *ad libitum*.

### 2.4. Extraction, Purification, and Quantification of P. Carinii

Six to eight weeks following inoculation, rats were euthanatized and parasite extraction was performed as previously described [34]. Briefly [34,35], parasites were extracted in Dulbecco's Modified Eagle's Medium (DMEM; BioWhittaker, Hyères, France) by agitation of lung pieces with a magnetic stirrer. The resulting homogenate was poured successively through gauze, 250 and 63 µm stainless steels filters. After centrifugation, the pellet was resuspended in a haemolytic buffered solution. *P. carinii* organisms were collected by centrifugation and then purified on a polysucrose gradient (Histopaque-1077, Sigma-Aldrich). Blood and Sabouraud dextrose agar (Difco, Pessac, France) media were inoculated with purified parasites to check for the presence of contaminating pathogens. *P. carinii* was quantitated on air dried smears stained with RAL-555 (Réactifs RAL, Martillac, France), a rapid panoptic methanol-Giemsa-like stain, which stains trophic forms, sporocytes, and cysts [30,35,36]. *P. carinii* was then cryopreserved by placing parasites in fetal calf serum with 10% dimethyl sulfoxide (DMSO) at −80 °C in a Nalgene 1 °C cryo freezing container (cooling rate: about 1 °C/min) for 4 hours [37]. The parasite samples were then stored in liquid nitrogen. Cryopreserved *P. carinii* were used for *in vitro* and *in vivo* studies.

### 2.5. Determination of the *in Vitro* Pneumocystis Activity

Drug stock solutions in DMSO (10 mg/mL) were diluted in Dulbecco's Modified Eagle's Medium (DMEM, BioWhittaker) supplemented with 10% heat-inactivated foetal calf serum (FCS, Gibco-BRL) to produce the required drug concentrations. *P. carinii* axenic short cultures were performed as follows. All the experiments were carried out in 24-well plates with a final volume of 2 mL of DMEM supplemented with 10% of heat-inactivated fetal calf serum (FCS) containing a final inoculum of 10^6^ organisms per mL. Plates were incubated for 4 days in an atmosphere of 5% CO_2_ at 37 °C. Then, *P. carinii* organisms were collected and quantified after RAL-555 panoptic staining. In parallel with these free-drug growth control wells, the anti-*Pneumocystis* effect of pentamidine and the other bisbenzamidines considered in this study was investigated in the same conditions. All molecules have been screened at a high (50 µg/mL), a medium (10 µg/mL), and a low (0.1 µg/mL) concentration. All experiments were performed in triplicate and data were statistically processed using Student’s t-test. Two-sided p-values < 0.05 were deemed to be statistically significant.

The concentration-effect relationship was established for **1**, **6**, and **7** by using the Hill equation: E_R_ = E_R,max_ X C^S^/[(EC_50_)^S^ + C^S^] where E_R_ is the effect of each drug concentration (C) on the percentage of inhibition estimated from experimental results; S is a parameter reflecting the steepness of the concentration-effect relationship curve; EC_50_ is the concentration of the compound at which 50% of the maximum effect (E_R,max_) is obtained. The parameters of this pharmacodynamic model were calculated by nonlinear least-squares regression techniques using commercial software (Sigma Plot, Systat Software Inc., San Jose, CA, USA).

### 2.6. Determination of the *in Vivo* Pneumocystis Activity

All animal experiments were performed following the guidelines of the Institut Pasteur de Lille animal study board, which conforms to the Amsterdam Protocol on animal protection and welfare, and Directive 86/609/EEC on the Protection of Animals Used for Experimental and Other Scientific Purposes, updated in the Council of Europe’s Appendix A (http://conventions.coe.int/ Treaty/EN/Treaties/PDF/123-Arev.pdf). The animal work also complied with the French law (nu 87-848 dated 19-10-1987) and the European Communities Amendment of Cruelty to Animals Act 1976. All experimental protocols involving animals were carried out by qualified personnel. The animal house (accreditation number: A59107, agreement number: B 59-350009) was placed under the direct control of the director of the Institut Pasteur de Lille who is the “designated responsible person” under French law. The study has been approved by the Ethical Committee for experiments on animals of the region Nord-Pas-de-Calais (approval number CEEA 022011).

To evaluate the *in vivo* anti-*Pneumocystis* activity, drug stock solutions in DMSO (300 mg/mL) were diluted in phosphate buffered saline (PBS, BioWhittaker) to produce the required final drug concentrations just before subcutaneous injection. Seven weeks p.i., animals were divided into groups of 4, and then pentamidine and derivatives **4** and **6** were dosed at 5 or 20 mg/kg by subcutaneous route. The drugs were given once a day for 10 consecutive days. The final concentration of DMSO in diluted drug solutions was maintained between 1.5% and 6%. Control animals received doses of 6%-DMSO in sterile water. Twenty-four hours after the end of the treatment, animals were euthanatized and the lung homogenised in a Stomacher-400 blender as previously described [37]. Quantitation of trophic forms, sporocytes, and cysts was performed on air-dried smears stained with RAL-555 stain [34,37,38]. Therapeutic efficacy was assessed by counting *P. carinii* parasites in lung homogenates and comparing them with those of the untreated controls at the end of the experiment. The results were expressed as percentage of inhibition versus drug-free animal controls and presented as mean and standard error. Data were analyzed using a two-way analysis of variance (PROC MIXED, SAS Institute, Cary, NC, USA), with sex, drug, and their interaction as independent predictors. Due to heterogeneity in parasites count distributions, the first step of the analysis was the selection of the within-subject covariance, based either on likelihood ratio tests or on the Bayesian Information Criterion. Then, tests of the fixed effects were performed. No interaction was found between sex and drug. Therapeutic efficacy was assessed with polynomial contrasts. Regression underlying assumptions were visually inspected with residual plots. Statistical significance was set at α = 0.05.

## 3. Results and Discussion

### 3.1. Chemistry

**Scheme 1:**
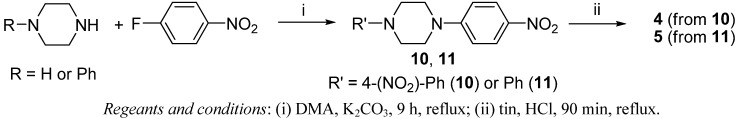
Procedure for the synthesis of the amine derivatives **4** and **5**.

**Scheme 2:**
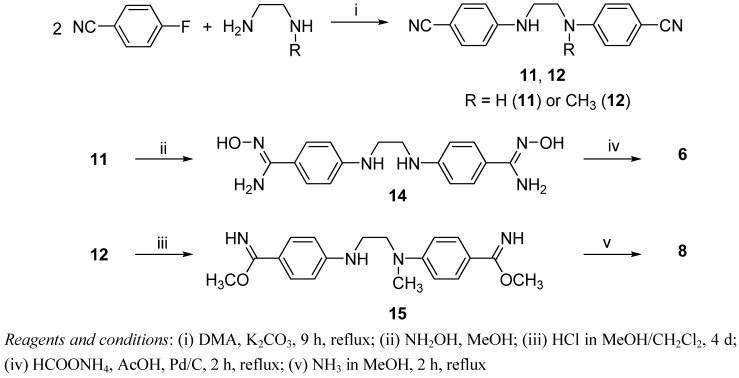
Procedures for the synthesis of bisbenzamidines **6** and **8**.

**Scheme 3:**
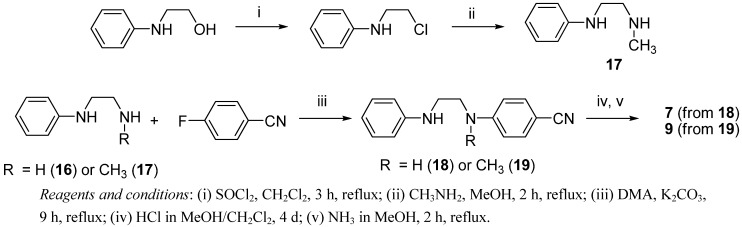
Procedures for the synthesis of the monobenzamidines **7** and **9**.

All compounds have been prepared according to established strategies depicted in Scheme 1, Scheme 2, Scheme 3. Reaction of piperazine or 4-phenylpiperazine with 4-fluoronitrobenzene afforded (Scheme 1) the corresponding nitro derivatives **10** and **11** (90% and 81% yield respectively) which were reduced to the amino compounds **4** (59% yield) and **5** (57% yield).

Three steps were required to synthesize the bisbenzamidines **6** and **8** (Scheme 2). The first one was common to both substances and consisted in the nucleophilic displacement of the fluorine atom in 4-fluorobenzonitrile by the amine function of an ethylenediamine derivative (70% yield for **11** and **12**). Depending on the nature of the central linker, the targeted amidines **6** (55% overall yield from **11**) and **8** (36% overall yield from **12**) were obtained either through reduction by ammonium formate in the presence of Pd/C of the amidoxime **14** or by a Pinner reaction.

Monobenzamidines **7** (36% yield from **16**) and **9** (10% yield from **17**) have been synthesized in a similar way (Scheme 3). In the case of the preparation of **9**, the precursor **17** has been obtained (10% overall yield), by converting *N*-phenylethanolamine into *N*-(2-chloroethyl)aniline with thionyl chloride, and further treatment with a methanolic solution of methylamine.

### 3.2. *In Vitro* Screenings

Cytotoxicity of derivatives **1**–**9** on the 3T6 murine embryonal fibroblast cell line was determined. For each compound, the % inhibition of activity against the concentration scale was plotted. The 50% inhibiting concentrations of cell proliferation (IC_50_) were calculated by locating the x-axis values corresponding to one-half of the absorbance values. The experiments were repeated in triplicate for each substance. Mean values are reported in Table 1. They ranged from 1.98 (**1** and **2**) to 56.06 µM (**3**). Interestingly, the diamidine **6** and the monoamidine **7** were significantly less toxic than pentamidine with IC_50_ values respectively 15- and 20-fold higher than for the reference compound. It should also be pointed out that *N*-methylation of the ethylenediamine linker afforded derivatives (**8**, **9**) characterized by a cytotoxicity similar to that of pentamidine.

In a first step the anti-*Pneumocystis carinii* susceptibility was determined at three concentrations: 50, 10, and 0.1 µg/mL. Derivatives bearing an amine function (compounds **4** and **5**) instead of an amidine were inactive even at the highest concentration (50 µg/mL *i.e.*, approximately 200 µM) . Despite the small size of the studied library, this confirms [19] the importance of the amidine moiety to express the antifungal activity. At the lowest concentration, 0.1 µg/mL *i.e.*, 0.17 µM, the reference drug, pentamidine **1**, was the most active compound still enabling to inhibit the fungus growth up to a level of 89%. Our data, again [39], emphasized the dramatic influence of the structure of the central linker separating the phamacophores. In fact, replacing the rigid central piperazine spacer in **2** and **3** by a flexible ethylenediamine chain (as in **6** and **7**) led to a significant increase of the anti-*Pneumocystis* effect, whichever the substance is a diamidine whether a monoamidine. Indeed at a concentration of 0.1 µg/mL (*i.e.*, in the range of 0.3 µM for the concerned substances), the piperazine-linked compounds **2** and **3** were less active than the corresponding linear derivatives **6** and **7**. On the other hand *N*-substitution in the ethylenediamine linker (to yield **8** and **9**) appeared to be detrimental and even rendered the monoamidine **9** inactive. As mentioned above, those two derivatives exhibited in addition a noticeable cytotoxicity towards the 3T6 cell line.

**Table 1:** Results of the *in vitro* cytotoxicity and anti-*Pneumocystis* screenings for derivatives **1**–**9**.

Compound	Cytotoxicity 3T6	Anti-*Pneumocystis* activity % inhibition *vs.* free-drug control at		
	IC_50_ (µM)	50 µg/mL	10 µg/mL	0.1 µg/mL*
**1** (pentamidine)	1.98 ± 0.20	99.0 ± 0.1	99.0 ± 0.1	88.9 ± 3.1
**2**	1.98 ±0.07	99.0 ± 0.1	99.0 ± 0.1	1.7 ± 2.9
**3**	56.06 ± 0.39	99.0 ± 0.1	99.0 ± 0.1	8.3 ± 14.4
**4**	40.8 ± 4.4	inactive	inactive	inactive
**5**	10.2 ± 0.6	inactive	inactive	inactive
**6**	30.24 ± 1.14	98.7 ± 0.6	97.7 ± 0.6	29.8 ± 19.7
**7**	40.60 ± 1.30	99.5 ± 0.4	98.7 ± 0.6	42.0 ± 12.5
**8**	2.48 ± 0.35	99.0 ± 0.0	99.0 ± 0.0	15.0 ± 12.5
**9**	3.11 ± 0.09	88.7 ± 2.5	86.3 ± 4.0	inactive

* 0.1 µg/mL corresponds to 0.17 (**1**); 0.26 (**2**); 0.32 (**3**); 0.27 (**6**); 0.28 (**7**); 0.26 (**8**); 0.33 (**9**) µM.

**Figure 2:**
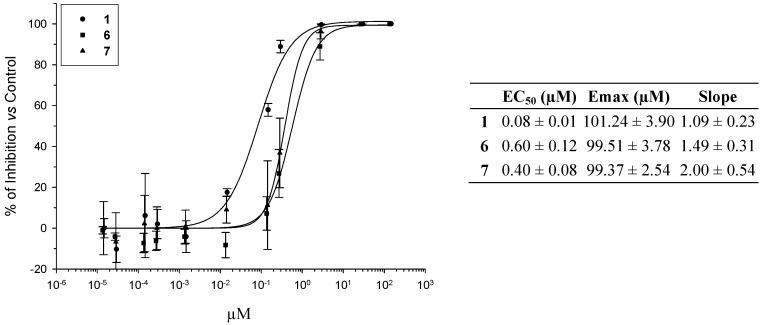
Concentration-*in vitro* activity relationships of pentamidine (**1**) and derivatives **6** and **7** against *P. carinii* (results were calculated after 4 days of culture).

Previous results [34] indicated that *in vitro* EC_50_ determination could be a valuable predictive indicator of the *in vivo* anti-*Pneumocystis carinii* pneumonia effect of a drug candidate. Therefore we selected pentamidine **1** and the two most active derivatives, **6** and **7**, to establish concentration-effect curves. Incubation time was fixed to 4 days and final drug concentrations range from 150 to 1.35 10^−5^ µM. Inspection of the results gathered in Figure 2 confirms the preliminary observations. Pentamidine **1** was the most potent drug with an EC_50_ value of 0.08 ± 0.01 µM (*p* < 0.05), followed by the monoamidine **7** (EC_50_ = 0.40 ± 0.08 µM, *p* < 0.05) and the diamidine **6** (EC_50_ = 0.60 ± 0.12 µM, *p* < 0.05). In terms of efficacy, all three derivatives exhibited similar optimum E_max_ values of 99.37 ± 2.54 µM (**7**), 99.51 ± 3.78 µM (**1**), and 101.24 ± 3.90 µM (**6**). In terms of concentration-effect relationships the steepness of the curves showed slight differences: S = 1.09 ± 0.23 for **1**, 1.49 ± 0.31 for **6**, and 2.00 ± 0.54 for **7**. Therefore the monoamidine **7** should be the most sensible to variations of concentrations.

### 3.3. *In Vivo* Study

**Figure 3:**
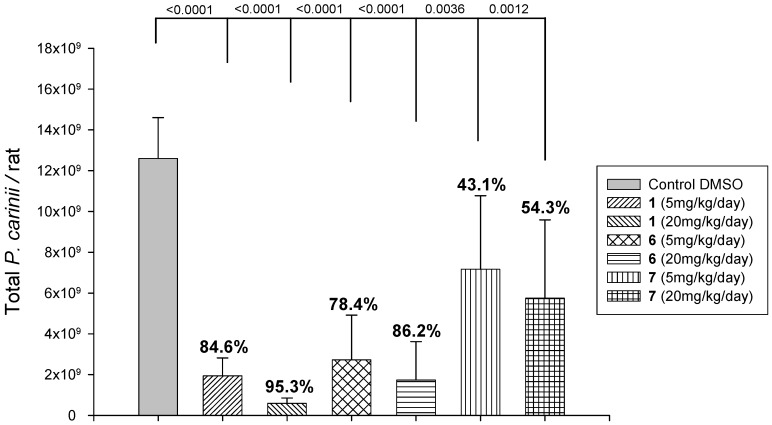
Therapeutic efficacy of pentamidine (**1**) and derivatives **6** and **7** against experimental *Pneumocystis carinii* pneumonia in nude rats (the numbers at the top of each bar graph indicate the % of inhibition *versus* control rats).

An *in vivo* experiment was performed in order to explore whether it reflected *in vitro* results. The used *in vivo* model was the athymic *Pneumocystis*-free Lou nu/nu rats non-surgically inoculated by endo-tracheal route [29,30] with a suspension of *P. carinii* organisms [32]. This model did not present the drawbacks of conventional corticosteroid-induced PcP rodent models [30], which are still used however. Rats developed high infection levels 6–8 weeks post inoculation without secondary fungal or bacterial infection. The non-surgical endotracheal inoculation method revealed to be simple, reliable, reproducible, and animal welfare compliant. Thus animals were endotracheally infected after 2 weeks of dexamethasone administration (1 mg/L, drinking water) with 10^7^* P. carinii* organisms. Drugs were administered once a day by subcutaneous route for 10 consecutive days. Untreated animals developed extensive infections at the end of the treatment period. The number of total *P. carinii* organisms per lung in DMSO control rats was 1.26 ± 0.20 × 10^10^. As illustrated in Figure 3, derivative **6** showed a strong anti-*Pneumocystis* activity comparable to that of pentamidine at both doses. On the other hand, whereas showing a strong anti-*Pneumocystis* activity *in vitro*, the monoamidine **7** appeared to be poorly potent when administered subcutaneously to *Pneumocystis*-infected rats, even at the dose of 20 mg/kg/day. That observation could be related to results [40] suggesting that the monoamidine **7** is more rapidly eliminated from plasma in comparison with pentamidine and the diamidine **6**.

## 4. Conclusions

Previous work [29] identified 4,4′-(1,4-piperazinediyl)bisbenzamidine (**2**) as an interesting anti-*Pneumocystis carinii* agent. We now have demonstrated that replacing the piperazine moiety by a flexible ethylenediamine chain yielded a novel promising hit, namely *N*,*N′*-bis(benzamidine-4-yl)ethane-1,2-diamine (**6**), characterized by a significant efficacy against *P. carinii in vitro* as well as *in vivo*. That derivative **6** exhibited a lower *in vitro* toxicity than pentamidine and was more efficient *in vivo* than the corresponding monoamidine **7**. Visual observations also indicated that rats treated with **6** maintained a quite normal behavior during the treatment and, last but not least, those rats did not develop any cutaneous reaction at the site of injections whereas deleterious wounds could be seen on the skin of the animals treated with pentamidine (data not shown).

Although genomic and phenotypic host species-related differences have been reported among *Pneumocystis* species, yielding a strong host-species specificity [1,36], it has not been established whether *Pneumocystis* species from different mammal hosts can exhibit different drug susceptibility patterns. The topic was rarely addressed but until now results obtained with *P. carinii in vitro* or *in vivo* could be extrapolated successfully to *P. jirovecii* in humans [41]. Therefore, **6**, the hit identified in this work, deserves further studies, especially as a potential candidate for the treatment of *Pneumocystis carinii* pneumonia cases.
